# Bacterial Pesticidal Protein Mpp51Aa1 Delivered via Transgenic Citrus Severely Impacts the Fecundity of Asian Citrus Psyllid, Diaphorina citri

**DOI:** 10.1128/aem.00723-23

**Published:** 2023-07-17

**Authors:** Ruchir Mishra, Ravishankar Narayana, Freddy Ibanez, Diann Achor, Turksen Shilts, Choaa El-Mohtar, Vladimir Orbović, Lukasz L. Stelinski, Bryony C. Bonning

**Affiliations:** a Entomology and Nematology Department, University of Florida, Gainesville, Florida, USA; b Citrus Research and Education Center, University of Florida, IFAS, Lake Alfred, Florida, USA; Danmarks Tekniske Universitet The Novo Nordisk Foundation Center for Biosustainability

**Keywords:** *Bacillus thuringiensis*, pesticidal proteins, Mpp51Aa1, Cry51Aa1, *Diaphorina citri*, Asian citrus psyllid

## Abstract

The Asian citrus psyllid (ACP) Diaphorina citri vectors the causative agent of citrus greening disease that has the capacity to decimate citrus production. As an alternative and more sustainable approach to manage D. citri than repeated application of chemical insecticides, we investigated the potential use of the bacteria-derived pesticidal protein, Mpp51Aa1, when delivered by transgenic Citrus sinensis cv. Valencia sweet orange or Citrus paradisi cv. Duncan grapefruit. Following confirmation of transcription and translation of *mpp51aa1* by transgenic plants, no impact of Mpp51Aa1 expression was seen on D. citri host plant choice between transgenic and control Duncan grapefruit plants. A slight but significant drop in survival of adult psyllids fed on these transgenic plants was noted relative to those fed on control plants. In line with this result, damage to the gut epithelium consistent with that caused by pore-forming proteins was only observed in a minority of adult D. citri fed on the transgenic Duncan grapefruit. However, greater impacts were observed on nymphs than on adults, with a 40% drop in the survival of nymphs fed on transgenic Duncan grapefruit relative to those fed on control plants. For Valencia sweet orange, a 70% decrease in the number of eggs laid by adult D. citri on transgenic plants was noted relative to those on control plants, with a 90% drop in emergence of progeny. These impacts that contrast with those associated with other bacterial pesticidal proteins and the potential for use of Mpp51Aa1-expressing transgenic plants for suppression of D. citri populations are discussed.

**IMPORTANCE** Pesticidal proteins derived from bacteria such as Bacillus thuringiensis are valuable tools for management of agricultural insect pests and provide a sustainable alternative to the application of chemical insecticides. However, relatively few bacterial pesticidal proteins have been used for suppression of hemipteran or sap-sucking insects such as the Asian citrus psyllid, Diaphorina citri. This insect is particularly important as the vector of the causative agent of citrus greening, or huanglongbing disease, which severely impacts global citrus production. In this study, we investigated the potential of transgenic citrus plants that produce the pesticidal protein Mpp51Aa1. While adult psyllid mortality on transgenic plants was modest, the reduced number of eggs laid by exposed adults and the decreased survival of progeny was such that psyllid populations dropped by more than 90%. These results provide valuable insight for potential deployment of Mpp51Aa1 in combination with other control agents for the management of D. citri.

## INTRODUCTION

A primary component of the integrated management of citrus greening disease, or huanglongbing, is the reduction of populations of the Asian citrus psyllid (ACP), Diaphorina citri, which vectors the presumed causative disease agent, “*Candidatus* Liberibacter asiaticus” (1). As D. citri is highly efficient at transmitting “*Ca.* Liberibacter asiaticus,” management of the vector has to be combined with other mitigation strategies, such as the direct targeting of “*Ca.* Liberibacter asiaticus” for effective mitigation of the disease caused by this pathogen (2).

While the use of insect-resistant transgenic plants is a theoretical option for psyllid management (3), there are relatively few examples of pesticidal protein-mediated resistance to hemipteran pests, with a few notable exceptions (4, 5). We and others (6) identified several bacterium-derived pesticidal proteins that are toxic to D. citri, including Mpp51Aa1 (formerly Cry51Aa1 [7]), and Cry1Ba1 (8, 9), both of which are derived from isolates of Bacillus thuringiensis. While the bacterium B. thuringiensis itself is used extensively for management of mosquitoes and pests in organic agriculture, for example (10), transgenic plant delivery of specific pesticidal proteins is the most effective approach to target sap-sucking pests such as D. citri. We therefore sought to investigate the impacts of these proteins on psyllids when delivered via transgenic citrus.

The product of any transgene introduced into citrus plants for control of D. citri needs to be expressed in phloem companion cells, and the resulting protein or RNA released into the sieve elements for acquisition by the psyllid. While secretion from the companion cell may be the default pathway in some cases, the mechanisms mediating secretion are not well understood. The successful expression of Cry1Ba1 with a secretion signal in three different transgenic citrus varieties and in a trap plant (Indian curry leaf plant; Bergera koenigii) resulted in adult psyllid mortality that appeared to correlate with the susceptibility of each plant variety to psyllid feeding (11).

Having demonstrated the toxicity of Mpp51Aa1 to adult and nymph D. citri (9), we sought to assess the potential for use of transgenic citrus expressing this pesticidal protein for management of D. citri populations. We assessed expression of the pesticidal protein with and without a signal peptide for enhanced delivery to the sieve element of the phloem, the site of psyllid feeding. Having confirmed transcription and translation of Mpp51Aa1 in transgenic Duncan and Valencia plants, we assessed the impacts of these plants on host plant preference, adult and nymph survival, and the production of progeny, all relative to wild-type (WT) plants. Significant impacts were observed, particularly on the progeny of exposed adult psyllids, suggesting that such plants have the potential for suppression of psyllid populations. The results of this study are discussed in the context of an optimal approach for use of bacterial pesticidal proteins in suppression of psyllid populations, with consideration given to sustainable use and resistance management.

## RESULTS

### Citrus transformation.

**FIG 1:**
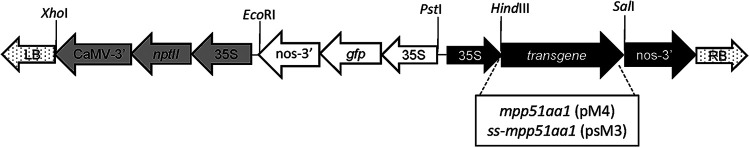
T-DNA from pCAMBIA2301-based binary vector used for transformation of Citrus paradisi cv. Duncan and C. sinensis cv. Valencia. Constructs were made for production of plants with (psM3 vector) and without (pM4 vector) the 23-amino-acid GNA secretory signal (ss), with transcription driven by the CaMV 35S promoter for expression in all plant tissues.

Valencia sweet orange and Duncan grapefruit were transformed for expression of *mpp51aa1* (codon optimized for efficient *in planta* expression), *nptII*, and *gfp*, with antibiotic resistance used as the basis for selection of transformants (Fig. 1; see Fig. S1 in the supplemental material). Green fluorescence was used to visualize protein expressed in transformants and to determine the rate of transformation in each case (Table 1). Between 19,000 and 37,000 shoots and buds that sprouted from Duncan and Valencia explants were inspected for fluorescence, with transformation rates of 0.44% and 0.59% for psM3 and pM4 Duncan, respectively, and 0.19% and 0.16% for psM3 and pM4 Valencia, respectively. The presence of *mpp51aa1* in transgenic plants was assessed with the 1:1:1 ratio of quantitative PCR (qPCR) threshold cycle (*C_T_*) values amplified with three different primer sets from the same DNA sample (Tables S1 and S2), indicating that the full-length *mpp51aa1* was present in the transfer DNA (T-DNA).

**TABLE 1:** Transformation rate for citrus cultivars used in this study

Cultivar	No. of shoots and buds inspected	No. of GFP-positive shoots and buds	Transformation rate (%)
Duncan			
psM3	28,907	127	0.44
pM4	19,488	115	0.59
Valencia			
psM3	37,184	70	0.19
pM4	34,009	56	0.16

### Confirmation of *mpp51aa1* transcription and translation.

Transcript levels of *mpp51aa1* were determined with reference to *actin* transcript levels for Duncan and Valencia plants generated using the psM3 and pM4 vectors. In some plants such as Duncan 552-03, *mpp51aa1* transcripts were not detected despite high green fluorescent protein (GFP) fluorescence, with variable levels of transcript accumulation in other plants (Table 2 and Table S3).

**TABLE 2:** Confirmation of transcription and translation for plants transformed for expression of Mpp51Aa1^*a*^

No.	Duncan	Transcript level^*b*^	Protein expression^*c*^	Valencia	Transcript level	Protein expression
1	543-05	5,391.44	−	375-01	9,777.74	+
2	**552-01**	379.1	+d	376-01	1,653.07	−
3	552-02c	89.7	+	376-02	2.97	−d
4	**553-01**	235.45	+	383-01	8,018.41	+
5	553-10c	32.6	−d	**392-01**	3,426.89	+
6	553-13	7,202.45	+d	393-05	1,211.33	−
7	555-03c	1,899.12	−d	396-01	239.2	−
8	556-11c	3,484.96	+	**396-02**	5,700.32	+d
9	556-12c	7,432.05	+	**398-01**	7,574.36	+
10	557-05	2,021.04	−d	**400-01**	2,789.56	+
11	**557-08**	2,274.93	+d	400-03	1,300.9	+
12	557-14	960.12	−d	**401-05**	549.6	+
13	557-16	3,948.86	−	**402-02**	6,454.77	+d
14	**558-02**	693.36	+d	**402-04**	6,078.44	+
15	559-15	113.48	−d	**402-05**	4,568.49	+
16	569-01	8,169.41	+	402-06c	76.9	−
17	569-02	1,451.6	+d	**409-01**	4,706.43	+
18	569-03	6,655.09	−d	WT	0	−
19	569-12c	125.54	−d	C4	0	−
20	570-01	11.5	−d	C6	0	−
21	570-02	2,896.9	+d			
22	**570-05**	6,695.05	+d			
23	570-06	2,267.98	+d			
24	570-07	188.56	−d			
25	570-08	4,893.85	+d			
26	551-07	92.3	+d			
27	551-09	2.9	−d			
28	551-11	177.2	−			
29	552-03	0	−			
30	553-04	37.3	−d			
31	WT	0	−			
32	E1-21 X-3	0	−			

a Accumulated transcript levels of *mpp51aa1* relative to actin and the presence of Mpp51Aa1 protein as determined by Western blot analysis in pM4 transgenic Duncan and Valencia plants are shown. Plants in bold were used for bioassays. Plants underlined were used for TEM analysis of psyllid guts.

b Variable levels of transcript accumulation were detected in all but one (552-03) of the transgenic plants for which GFP expression was confirmed.

c Mpp51Aa1 expression was confirmed for all but three Duncan and four Valencia plants for which transcription was confirmed. This result highlights that translation cannot be assumed if transcription is confirmed. +, protein expression confirmed; −, no protein of the expected size detected; blank, not assessed; d, Mpp51Aa1-immunoreactive bands at a smaller size than expected are present, indicative of degradation. Western blot analysis is provided in the supplemental material.

Western blot analysis was conducted on leaf petioles, veins, and midribs to test for expression (positive or negative) of Mpp51Aa1 in transgenic lines and not to compare protein levels between lines. The full-length Mpp51Aa1 of 35 kDa was only detected in transgenic Duncan and Valencia produced using pM4, i.e., without inclusion of the snowdrop lectin, Galanthus nivalis agglutinin (GNA)-derived secretory signal peptide (Table 2; Fig. S2). Full-length Mpp51Aa1 (without the GNA signal) was detected in a total of 15 Duncan plants and 12 Valencia plants (Table 2). In contrast, expression of Mpp51Aa1 with the GNA signal (from the psM3 vector) resulted in degradation of Mpp51Aa1 in some cases, with degradation products of ~16 to 17 kDa apparent in the Western blots (Fig. S2; Table S3). As no full-length Mpp51Aa1 with the GNA signal was detected in Duncan or Valencia plants transformed with psM3, these plants were not used for subsequent assessment of impacts on D. citri.

### The effect of Mpp51Aa1 expression in Duncan grapefruit on D. citri plant preference.

Behavioral assays conducted to assess whether Mpp51Aa1 expression impacted psyllid host selection showed no preference between wild-type and transgenic Duncan plants (Fig. S3). Approximately 70% of the psyllids settled on one of the two plants and with equal frequency on transgenic and wild-type plants (chi-square test = 0.8554; degrees of freedom (*df*) = 1; *P* = 0.357862).

### Impact of transgenic Duncan plants on D. citri survival.

**FIG 2:**
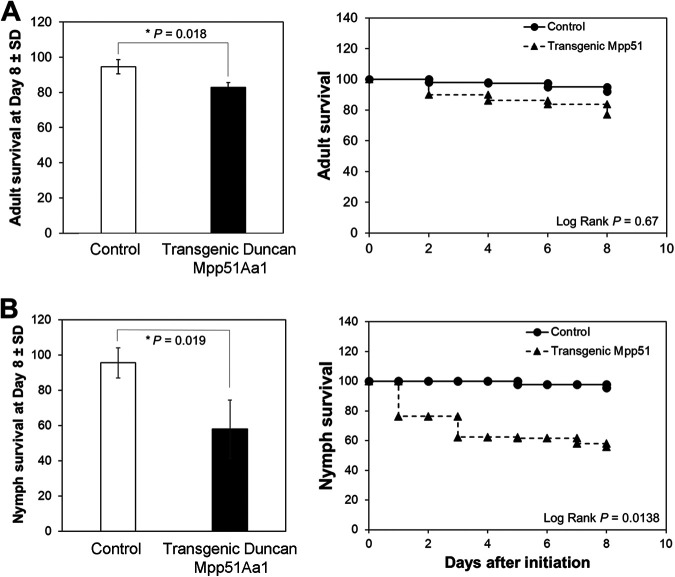
Transgenic Citrus paradisi cv. Duncan expressing Mpp51Aa1 reduced survival of adult and nymph Diaphorina citri. Bars represent the mean Asian citrus psyllid (ACP), D. citri, adult (A) and nymph (B) survival ± standard deviation (SD), with statistical differences indicated (log-rank test for pairwise comparisons). Adult (A) or nymph (B) D. citri survival during the course of the bioassay is also shown at right for one of three independent replicates per treatment. Curves depict the Kaplan-Meier survival probabilities of D. citri after access to transgenic plants.

Whole-plant bioassays demonstrated a slight but significantly reduced survival (by ~10%) of adult psyllids maintained on Mpp51Aa1-expressing transgenic Duncan plants relative to those on wild-type plants by day 8 (*P* < 0.05) (Fig. 2). A total of ~400 psyllids were in continuous contact with plants during the course of this bioassay, with five Duncan plants tested in each of four biological replicates. A greater impact of these transgenic plants was seen for nymphs in detached leaf assays (*P* < 0.05), with about 50% of nymphs succumbing to Mpp51Aa1 exposure.

When vegetative growth (presence/absence of feather flush) was considered a possible variable affecting feeding and subsequent survival of adult D. citri on Mpp51Aa1 transgenic Duncan plants, no statistical differences were observed from nonflushing plants (Fig. S4). Mpp51Aa1 transgenic Duncan plants that exhibited high feather flush intensity did not cause greater D. citri adult mortality than nonflushing plants.

On examination by transmission electron microscopy (TEM) of up to 20 sections per gut of adult D. citri maintained for 8 days on Mpp51Aa1-expressing Duncan (*n* = 9) or control (*n* = 7) plants, anomalies in the structure of the microvilli were observed in a subset of samples from the transgenic but not the wild-type plants (Fig. S5). While the microvilli that line the psyllid midgut of insects maintained on control plants were full and typically well ordered, those of ~20% of the insects maintained on transgenic plants were thin, short, and sometimes disrupted. Observation of damage in only a subset of insects is consistent with the relatively low impact of Duncan-expressed Mpp51Aa1 on the survival of adult psyllids.

### Impact of transgenic Valencia sweet orange on D. citri adult and progeny survival.

**FIG 3:**
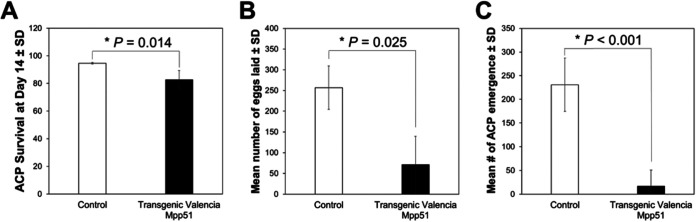
Transgenic Citrus sinensis cv. Valencia expressing Mpp51Aa1 reduced survival and fecundity of adult Diaphorina citri. Impact of transgenic C. sinensis cv. Valencia expression of Mpp51Aa1 on adult psyllid survival (A) at day 14, the number of eggs laid (B), and the emergence of progeny (C) compared to impacts on control plants. Bars represent standard deviation (SD) with statistical differences indicated (log-rank test for pairwise comparisons).

Similar to results for transgenic Duncan plants, the long-term bioassay on transgenic Mpp51Aa1 Valencia plants showed a modest but significantly reduced survival of adult psyllids compared with that observed on controls (*P* < 0.05) (Fig. 3). A dramatic drop was seen, however, in the number of eggs laid (~70 compared to ~250 on control plants; *P* = 0.025) and in the number of progeny adult psyllids, with ~225 on control plants compared to ~10 on transgenic plants (*P* < 0.001) (Fig. 3).

## DISCUSSION

The goal of this study was to examine the impact of Mpp51Aa1 on D. citri when delivered via transgenic citrus plants, specifically Duncan grapefruit and Valencia sweet orange. While the impact of plant-expressed Mpp51Aa1 on adult psyllids was modest, greater mortality was seen for nymphs, consistent with the increased susceptibility of nymphs to this protein relative to that of adult D. citri (9). The greatest impact was seen, however, on the progeny, with a massive reduction in the number of eggs laid by adult psyllids fed on transgenic Valencia plants and subsequent emergence of the F1 generation relative to control plants. These results suggest that D. citri populations could be reduced by 90% or more within two generations by Mpp51Aa1-expressing transgenic citrus.

The higher transformation rate for grapefruit than sweet orange is consistent with a previous report for transgenic expression of Cry1Ba1 in citrus (11). Several observations made for transgenic plants expressing Mpp51Aa1, however, contrasted with the expression of Cry1Ba1 in citrus (11, 12). First, the use of a signal peptide to promote transport into the sieve element resulted in the apparent instability of expressed Mpp51Aa1, which was not the case for Cry1Ba1. Second, the transgenic citrus plants expressing Mpp51Aa1 did not exhibit the delayed wilting phenotype that was characteristic of Cry1Ba1 expression (11, 12), and third, damage to the gut epithelium reflecting the physiological damage caused by Mpp proteins (13) was only seen in a subset of Mpp51Aa1-exposed adults, consistent with the relatively low adult mortality rate.

The ETX/MTX (Mpp) structural class of bacterial pesticidal proteins are beta pore-forming proteins with similar impacts on targeted pests to those caused by other bacteria-derived pesticidal proteins (14, 15). Several factors could account for the relatively low mortality observed for adults, with almost total loss of the F1 generation. First, stress and reduced feeding associated with sublethal exposure to Mpp51Aa1 could reduce resources essential for the survival of progeny. While adult psyllids did not discriminate between transgenic and control plants in choice test assays, Mpp51Aa1-mediated damage to the gut following the onset of feeding could result in cessation of feeding by adult psyllids with resulting diminished resources for reproduction. Second, feeding inhibition would have greater impacts on nymphs due to their limited mobility: winged adults are able to readily change their feeding site, while nymphs are localized to the site of maternal egg laying. Adults could therefore move away from plants expressing Mpp51Aa1, while nymphs cannot. The electrical penetration graph system could be used to monitor psyllid feeding to assess whether adult feeding stops on Mpp51Aa1 transgenic plants following exposure to the pesticidal protein (16). Moreover, in contrast to adult psyllids that can survive without feeding for 5 days, nymphs only survive for 48 h. Third, nymphs may receive a larger overall dose of Mpp51Aa1 over a longer period of sustained and uninterrupted feeding while undergoing development than do adults. The greater impact on nymphs than on adults aligns with the greater susceptibility of nymphs relative to adults to Mpp51Aa1 (9) and with the impacts of the modified Mpp51Aa2 on *Lygus* nymph development (13).

For Cry1Ba1, significant differences were seen in adult psyllid survival on transgenic Valencia, but not on transgenic Duncan plants, relative to the respective control plants (11). In contrast, significant differences in adult mortality were seen in both cases on expression of Mpp51Aa1, with the same bioassay methodology used for generation of day 14 mortality data. This difference could result from differences in pesticidal protein expression levels in plants used for bioassays, particularly for Cry1Ba1 expression in transgenic Duncan plants as previously discussed (11). However, in transmission electron micrographs, damage to the gut epithelium was only noted for a small proportion of adults fed on Mpp51Aa1 transgenic Duncan, in sharp contrast to consistent damage observed for psyllids fed on Cry1Ba1-expressing plants (11). This result indicates that insufficient Mpp51Aa1 in its active form was acquired by adult psyllids in the current study.

The 50% lethal concentration (LC_50_) of Cry1Ba1 for adult D. citri at 125 μg/mL (8) is comparable to that of Mpp51Aa1 at 110 μg/mL (9). However, adult mortality data and TEM results showing damage to the gut epithelium are consistent with greater toxicity of plant-delivered Cry1Ba1 to adult D. citri, with considerably more damage to the gut epithelium than insects maintained on transgenically expressed Mpp51Aa1. One potential explanation for the discrepancy between LC_50_ data and observed mortality and tissue damage resulting from *in planta* expression between the two pesticidal proteins is that a greater portion of the expressed Cry1Ba1 was delivered to the sieve element by the GNA secretion peptide for acquisition by D. citri, relative to Mpp51Aa1. Expression of Mpp51Aa1 with the signal peptide (psM3 construct) resulted in protein instability, with little full-length Mpp51Aa1 detected in Western blots (see Fig. S2 and Table S3 in the supplemental material). Bioassays were therefore only conducted with plants expressing Mpp51Aa1 without the signal peptide (pM4 construct). While the closely related pesticidal protein Mpp51Aa2 was expressed in cotton without a signal peptide for insect resistance, the feeding of the targeted pests, *Lygus* spp., is not phloem limited (4). In the event that the delivery of Mpp51Aa1 to the sieve element is limiting for protein acquisition by D. citri, optimization of the psM3 construct, including the use of alternative secretion peptides that do not impact protein stability, will be warranted.

Proteins synthesized by companion cells in the range of 20 to 60 kDa are transferred via plasmodesmata to sieve elements in the phloem of multiple plant species (17). It has been suggested that larger proteins are unfolded prior to transport, with unfolding mediated via chaperones, and that the relative distribution of a given protein between the companion cell and sieve element relates to the efficiency of transport between the two compartments (18). While proteins of 3 to 20 kDa may passively diffuse through the pore-plasmodesma units, the mechanisms underlying the movement of larger proteins are unclear (19). Specific structural motifs, including N-terminal residues, are known to be required in some cases (20, 21). Whether or not a signal sequence such as that derived from GNA is needed for bacterial pesticidal proteins to localize to the sieve element may depend on the protein, with relative size being a primary consideration. Indeed, expression of the 71-kDa pesticidal protein Cry11Aa4 (22) in Hamlin and Valencia (Citrus sinensis L. Osbeck) without a signal peptide resulted in <44% D. citri nymph mortality after 5 days (23). This relatively low level of nymph mortality could result from inefficient transport of Cry11Aa4 from the companion cell into the sieve element in the absence of a signal peptide.

An alternative scenario for the lower-than-expected adult psyllid mortality on Mpp51Aa1-expressing transgenic plants relates to whether Mpp51Aa1 is available to the psyllid in its most toxic form. This protein produces a 68-kDa dimer (15), with the dimer being less toxic than monomeric Mpp51Aa1 (13). The dimeric protein is processed by removal of C-terminal amino acids by trypsin (present in the saliva of some hemipteran species) into the monomeric form, which has greater gut binding affinity than the dimer. The high level of protein expression driven by the 35S promoter and concentration of Mpp51Aa1 in the companion cell could promote dimer formation such that transfer to the sieve element is reduced. Alternatively, the presence of the dimer in the sieve element would confer a lower level of insect resistance than presence of the monomer.

Our results indicate that pesticidal proteins with greater toxicity against adult psyllids may be required if the goal is to achieve high adult mortality. However, we previously demonstrated high mortality of adult D. citri on expression of Cry1Ba1 from a transgenic trap plant, Bergera koenigii (11). The coexpression of Cry1Ba1 and Mpp51Aa1 in such trap plants may result in even greater population suppression. The combination of pesticidal proteins that differ in their mode of action can delay the onset of resistance in the targeted insect (24), and the progeny of psyllids that survive exposure to Cry1Ba1 would be significantly impacted by Mpp51Aa1. The use of such pesticidal protein combinations for pest suppression parallels the use of B. thuringiensis itself in pest management, which harbors plasmids encoding multiple pesticidal proteins. As an example, B. thuringiensis subsp. *israelensis* has been successfully applied for control of the aquatic larvae of mosquitoes for many years without resistance in the field (25).

### Considerations for use of transgenic plants in citrus production.

Resistance management is a primary consideration for the use of transgenic plants expressing pesticidal proteins (26). There are several important components for effectively delaying resistance, which are (i) plant expression of a high dose sufficient to kill insects that are heterozygous for the resistance gene, (ii) availability of a refuge where the target species can reproduce in the absence of selection pressure to maintain susceptible insects in the population, (iii) consideration of the behavior of the targeted pest species as sufficient dispersal for mating with susceptible insects from the refuge is required, and (iv) a fitness cost associated with the resistance phenotype. In the case of D. citri, long-distance migration is relatively unusual (27, 28), and under favorable conditions, dispersal is limited at <15 m (29). Based on this limited dispersal behavior, groves planted entirely with transgenic citrus may be suboptimal. While plants attractive to psyllids that offer a refuge could be planted within a grove, it is unlikely that citrus growers would be open to this approach. Citrus growers have experience with planting windbreaks around groves to decrease disease transmission (30, 31) and would be amenable to planting a border row of noncrop plants that serve as a cultural control method. Therefore, the use of a trap plant such as the Indian curry leaf plant, B. koenigii, which is more attractive to psyllids than fruit-bearing citrus varieties (32–34) but does not support “*Ca.* Liberibacter asiaticus” replication (35), becomes a particularly attractive option for psyllid management as previously proposed (11).

When the dispersal and feeding behaviors of D. citri, resistance management considerations, grower preferences, and public sentiment with respect to bioengineered foods are all taken into account, the approach that is most likely to be adopted for pesticidal protein-mediated psyllid management is use of a transgenic trap plant that expresses multiple agents that are toxic to D. citri. These trap plants would be planted around groves in conjunction with a “push-pull” strategy (36): the highly attractive Indian curry plants (“pull”) would be used in conjunction with a deterrent such as kaolin (37, 38) applied to the fruit-bearing citrus. This strategy would be used in conjunction with agents targeting “*Ca.* Liberibacter asiaticus” as part of an integrated approach for management of citrus greening.

Comprehensive analysis of the specificity of pesticidal proteins is required prior to their deployment for use in pest management, as specified by national regulatory agencies such as the U.S. Environmental Protection Agency. Activity of the modified Mpp51Aa2 used for *Lygus* spp. and thrips management in cotton is limited to three insect orders, with no activity against mammals, pollinators, or decomposers (39, 40). Low toxicity against the predatory pirate bug (*Orius* spp.) was found to be inconsequential due to minimal exposure under field conditions (41). Similar studies would be required for the use of any pesticidal protein in citrus groves. The potential impacts of the pesticidal protein on beneficial insects and natural enemies of D. citri, with exposure within the citrus grove ecosystem taken into consideration, are of particular importance.

In conclusion, this study demonstrates the impact of Mpp51Aa1 expressed by transgenic Duncan and Valencia plants on the progeny of exposed adult D. citri, with a >90% reduction in the F1 generation relative to control treatments. As low adult psyllid mortality is suboptimal from a resistance management perspective, further work is needed to determine (i) whether low acquisition of Mpp51Aa1 by psyllids results from low levels of active Mpp51Aa1 in the sieve elements and assessment of methods for optimization, (ii) impacts of Mpp51Aa1 and Cry1Ba1 in combination against D. citri, and (iii) whether additional pesticidal proteins with greater toxicity against D. citri can be identified.

## MATERIALS AND METHODS

### Vector construct and plant transformation.

The plasmid pHT304-Mpp51Aa1 containing the Mpp51Aa1 sequence (GenPept accession number ABI14444) was kindly provided by Michael J. Adang, University of Georgia, United States (15). *Agrobacterium* cultures were created with modified pCAMBIA2301-derived binary vectors psM3 and pM4 (Fig. 1) carrying citrus (grapefruit) codon-optimized *mpp51aa1* with and without a secretion signal (23 amino acids) derived from the snowdrop lectin, Galanthus nivalis agglutinin (GNA) (21), respectively (see Fig. S1 in the supplemental material). The constitutive CaMV 35S promoter was used for Mpp51Aa1 expression throughout the plant, including the phloem, the site of psyllid feeding (42, 43). These vectors also encode kanamycin resistance (neomycin phosphotransferase II [*nptII*]) and GFP to facilitate selection of transgenics. The psM3 and pM4 vectors were mobilized into Agrobacterium tumefaciens EHA105 competent cells. Duncan grapefruit (Citrus paradisi Macf.) and Valencia sweet orange (Citrus sinensis L. Osbeck) were transformed using methods as previously described (44). Briefly, etiolated seedlings of each cultivar were cut into 10- to 15-mm explants that were incubated in freshly prepared suspension of A. tumefaciens. Explants were then maintained for 2 days on cocultivation medium (CCM) (45) before transfer to regeneration medium (RM) for shoot induction and elimination of *Agrobacterium*. Shoots that sprouted from seedling explants after 5 weeks were inspected for green fluorescence resulting from GFP expression under a fluorescence stereomicroscope (Leica MZ16 FA; Leica, Bannockburn, IL, USA) with a 480-nm-excitation blue light. GFP-positive shoots were micrografted on Carrizo rootstock plants. These plantlets were transferred to 8-cm^3^ pots covered with transparent plastic domes to maintain high humidity and left with constant white light (fluence rate, 50 μmol/m^2^s). Plantlets were moved to the greenhouse 2 months later and transferred to larger pots.

The presence of *mpp51aa1* was confirmed by qPCR (46). Sample preparation with DNA extracted from single leaves of plants (up to 4 years old) and qPCR were conducted as described previously (12). qPCR was performed using three different primer pairs (Table S1) to amplify different parts of *mpp51aa1* in a 7500 thermal cycler (Applied Biosystems, Waltham, MA, USA) using Power SYBR green RNA-to-C_T_ 1 step kit (Applied Biosystems; catalog no. 4391178) without the reverse transcription (RT) step. The quality of DNA isolated from transgenic and control plants was confirmed by amplification of the *actin* gene using previously described primers. The qPCR volume of 10 μL was comprised of 5 μL 2× SYBR mix, 0.5 μL 10 mM forward primer, 0.5 μL 10 mM reverse primer, 0.1 μL SYBR enzyme, and 1 μL template DNA diluted 1:50 to negate the effect of PCR inhibitors present in the resuspended DNA. The program was run at 95°C for 10 min, 40 cycles of 95°C for 15 s, and 60°C for 1 min. A melting curve was run at the end of the qPCR program.

### Confirmation of transcription and translation from *mpp51Aa1*.

Transcript levels for *mpp51aa1* were assessed by RT-qPCR. The RNeasy plant minikit (Qiagen, Germantown, MD, USA) was used for the extraction of total RNA from the leaves of transgenic or wild-type (WT) citrus plants. cDNA synthesis from 1 μg of RNA was conducted using the iScript cDNA synthesis kit (Bio-Rad Labs, Hercules, CA, USA). A fragment of the actin transcript amplified using ACT primers (12) was used as a reference for normalization, and *mpp51aa1* transcripts were amplified using the primers RN3f (5′-TTGCTAATTGGCCTAATCTTCC-3′) and RN3r (5′-CACCAGCTGAAACTTGAGAAAC-3′). The iTaq universal SYBR green mix (Bio-Rad Labs) was used for PCR validations with the following program: 95°C for 2 min, 42 cycles at 95°C for 5 s, 55°C for 10 s, and 72°C for 15 s. The specificity of PCR amplification was verified by melt curve analysis (from 55°C to 95°C). The levels of *mpp51aa1* transcripts in transgenic plants relative to WT were calculated using the threshold cycle (2^−ΔΔ^*^CT^*) method (47).

To extract soluble proteins from wild-type plants or transgenic plants (1 to 3 years old) for detection of Mpp51Aa1 in Western blotting, 15 to 100 mg of leaf or stem tissue was pulverized with liquid nitrogen in a mortar and pestle. The powdered tissue was then solubilized overnight at 4°C in 500 μL of extraction buffer (50 mM Tris-HCl, pH 7.5, 10 mM MgCl_2,_ 150 mM NaCl, 0.1% IGEPAL CA-630, 1 mM phenylmethylsulfonyl fluoride [PMSF], and 1× protease inhibitor [cOmplete Mini EDTA-free protease inhibitor cocktail; Roche, Basel, Switzerland]) at 4°C. The solution was centrifuged at 16,100 × *g* for 15 min at 4°C. The soluble proteins were obtained from the aqueous supernatant layer, and the protein concentration was determined by Bradford assay (48). Solubilized protein samples, along with Mpp51Aa1 expressed in E. coli strain BMB171 and purified as described previously (9) (positive control), were separated in a 4 to 12% SDS-PAGE gel (Bio-Rad). Proteins were then transferred to a polyvinylidene difluoride (PVDF) membrane (Amersham International PLC, Amersham, UK). The membrane was blocked with 1× Tris-buffered saline (TBS), 0.1% Tween 20, and 5% bovine serum albumin (BSA). Mpp51Aa1 was detected with anti-Mpp51Aa1 (1:2,500) and a horseradish peroxidase (HRP)-coupled secondary antibody (Thermo Fisher Scientific, Waltham, MA, USA; dilution, 1:2,500) followed by a chemiluminescent substrate (Pierce, Thermo Fisher Scientific). The anti-Mpp51Aa1 antiserum was raised in rabbits (GenScript Biotech Corp., Piscataway, NJ, USA) using Mpp51Aa1 expressed and purified as described previously (9) as antigen.

### Impact of transgenic Duncan plants on D. citri.

Asian citrus psyllids, D. citri maintained in a greenhouse at the Citrus Research and Education Center (University of Florida, Lake Alfred, FL, USA), were used in bioassays. The colony is insecticide susceptible, free of “*Ca.* Liberibacter asiaticus,” and maintained on wild-type Duncan grapefruit (Citrus paradisi Macf.) or Valencia sweet orange (Citrus sinensis L. Osbeck) at 27 to 28°C, 60 to 65% relative humidity, and a 14:10-h (light/dark) photoperiod with a maximum photosynthetic radiation of 215 μmol s^−1^ m^−2^. Plants were watered twice per week and fertilized twice per month with an alternating schedule of a 24:8:16 NPK solution at 4 g L^−1^ (Miracle-Gro all-purpose plant food; Scotts Miracle-Gro Products, Marysville, OH, USA) and a 6:4:6 (NPK) granular fertilizer at 1 g per pot (Expert Gardener Gro Tec. Inc., Madison, GA, USA). Subsequent biological evaluations employed psyllids that had been reared on the same hosts as treatment plants to control for possible effects of host adaptation.

To assess the impacts of transgenic Duncan plants for which *mpp51aa1* transcription had been confirmed on ACP adults, bioassays were conducted using whole plants. Prior to use in bioassays, Duncan plant leaves were washed using a 2% Dawn dish detergent spray solution (Procter and Gamble, Cincinnati, OH, USA) twice per week. Plants were then rinsed with clean water 20 to 30 min after detergent was applied. The plants were then moved into individual cages under the conditions described above.

Treatment plants were transformed Duncan grapefruit plants as described above, and control plants were nontransformed wild-type plants of similar size, age, and variety. A total of 20 adult psyllids of mixed age and sex were released onto each plant (*n* = 5), and insect survival was scored every 48 h over a 192-h period. Insects found on their side or back that were unable to move upon prodding with a metal probe were considered dead. The bioassay was repeated four times. To determine whether the presence of flush impacted adult psyllid survival, the presence or absence of flush was monitored for each plant, and data were analyzed separately for comparison between the two conditions.

To assess the impact of transgenic Duncan plants on nymphs, bioassays were conducted using excised mature leaves placed into petri dishes (35 mm) using the methods described in Chen et al. (49). The survival of ACP nymphs in petri dish assays was assessed every 48 h for a total of 192 h.

To determine whether Mpp51Aa1 expression impacted host plant choice, the choice and settling behavior of newly emerged adult psyllids between transgenic Duncan grapefruit plants and control plants (*n* = 5) were evaluated with an open-air choice assay as described previously (50). Control plants were of the same approximate age and size as test plants. A total of 20 psyllids were introduced into the chamber, and the number of psyllids found in a characteristic feeding position (abdomen slanted at 45° angle to the leaf surface) on each plant was counted every 24 h for 5 days. The experiment was repeated twice. Psyllids tended to remain on the plant colonized initially, and therefore, data are presented cumulatively as host settling after 5 days of observation.

### Transmission electron microscopy of D. citri fed on transgenic Duncan and control plants.

Following Western blot confirmation of Mpp51Aa1 expression by transgenic plants, the impacts of transgenically expressed Mpp51Aa1 on the guts of D. citri were evaluated by TEM. Micrographs were used to examine the midgut epithelium of test and control psyllids fed on three transgenic or three wild-type Duncan plants (plants 553-01, 552-01, and 558-02). A total of 20 psyllids were maintained on the test or control plants for a period of 8 days. The psyllids from each treatment were then collected, pooled, and anesthetized on ice. The heads and tips of the abdomens were removed, and samples were fixed in 3% (vol/vol) glutaraldehyde in phosphate-buffered saline (PBS) buffer for 16 h at 4°C, postfixed in 2% (vol/vol) osmium tetroxide for 4 h at room temperature, dehydrated in acetone, and embedded in Spurr’s resin (51). A total of 9 to 10 psyllids per treatment were sectioned and examined by light microscopy for orientation purposes and to identify the midgut. Ultrathin 90-nm sections mounted on copper grids were stained with aqueous uranyl acetate (2%, wt/vol) and lead citrate (52) prior to observation using an FEI Morgagni 268 TEM (FEI Company, Hillsboro, OR, USA).

### Impact of transgenic Valencia plants on D. citri.

Given our initial observations that transgenic Duncan plants expressing Mpp51Aa1 had greater effects on nymphs than adults, we adjusted our bioassay method to more carefully discern possible transgenerational effects of Mpp51Aa1-expressing plants on psyllids. Bioassays were conducted on transgenic and control Valencia plants to assess not only the impact of exposure on adult psyllids but also on egg production and the subsequent F1 generation. Whole-plant assays were therefore conducted for 90 days. As before, on the day of initiation, 20 7- to 10-day-old D. citri adults of mixed sex were collected and transferred to each plant. Survival was scored every 48 h for the initial 14 days of the assay to record adult psyllid survival. On day 14, all surviving adults were removed, and total mortality of released adults was determined as had been done previously. Subsequently, the survival of F1 nymphs and adults emerging from eggs laid during the initial 14-day period was then determined until assay termination on day 90.

### Statistical analyses.

Bioassay data for adult or nymph survival on Mpp51Aa1 transgenic or control plants were analyzed using a Kaplan-Meier survival analysis (53), and a log-rank test for pairwise comparisons was performed using RStudio environment version 3.6.3 (54). Behavioral response by D. citri between the two host outcomes was analyzed using chi-square contingency tables, also in R (54).

### Data availability.

All data associated with this study are provided in the article and supplemental material.

## References

[1] Grafton-Cardwell EE, Stelinski LL, Stansly PA. 2013. Biology and management of Asian citrus psyllid, vector of the huanglongbing pathogens. Annu Rev Entomol 58:413–432. doi:10.1146/annurev-ento-120811-153542. 23317046

[2] Lee JA, Halbert SE, Dawson WO, Robertson CJ, Keesling JE, Singer BH. 2015. Asymptomatic spread of huanglongbing and implications for disease control. Proc Natl Acad Sci USA 112:7605–7610. doi:10.1073/pnas.1508253112. 26034273 PMC4475945

[3] Heckel DG. 2020. How do toxins from Bacillus thuringiensis kill insects? An evolutionary perspective. Arch Insect Biochem Physiol 104:e21673. doi:10.1002/arch.21673. 32212396

[4] Baum JA, Sukuru UR, Penn SR, Meyer SE, Subbarao S, Shi X, Flasinski S, Heck GR, Brown RS, Clark TL. 2012. Cotton plants expressing a hemipteran-active Bacillus thuringiensis crystal protein impact the development and survival of Lygus hesperus (Hemiptera: Miridae) nymphs. J Econ Entomol 105:616–624. doi:10.1603/ec11207. 22606834

[5] Mishra R, Arora AK, Jimenez J, Dos Santos Tavares C, Banerjee R, Panneerselvam S, Bonning BC. 2022. Bacteria-derived pesticidal proteins active against hemipteran pests. J Invertebr Pathol 195:107834. doi:10.1016/j.jip.2022.107834. 36244507

[6] Dorta SO, Balbinotte J, Monnerat R, Lopes JRS, da Cunha T, Zanardi OZ, de Miranda MP, Machado MA, de Freitas-Astua J. 2020. Selection of Bacillus thuringiensis strains in citrus and their pathogenicity to Diaphorina citri (Hemiptera: Liviidae) nymphs. Insect Sci 27:519–530. doi:10.1111/1744-7917.12654. 30548193

[7] Crickmore N, Berry C, Panneerselvam S, Mishra R, Connor TR, Bonning BC. 2021. A structure-based nomenclature for Bacillus thuringiensis and other bacteria-derived pesticidal proteins. J Invertebr Pathol 186:107438. doi:10.1016/j.jip.2020.107438. 32652083

[8] Fernandez-Luna MT, Kumar P, Hall DG, Mitchell AD, Blackburn MB, Bonning BC. 2019. Toxicity of Bacillus thuringiensis-derived pesticidal proteins Cry1Ab and Cry1Ba against Asian citrus psyllid, Diaphorina citri (Hemiptera). Toxins (Basel) 11:173. doi:10.3390/toxins11030173. 30909400 PMC6468527

[9] Tavares CS, Bonning BC. 2022. Mpp51Aa1 toxicity to Diaphorina citri nymphs demonstrated using a new, long-term bioassay method. J Invertebr Pathol 195:107845. doi:10.1016/j.jip.2022.107845. 36270336

[10] Lacey LA, Grzywacz D, Shapiro-Ilan DI, Frutos R, Brownbridge M, Goettel MS. 2015. Insect pathogens as biological control agents: back to the future. J Invertebr Pathol 132:1–41. doi:10.1016/j.jip.2015.07.009. 26225455

[11] Orbović V, Ravanfar SA, Achor DS, Shilts T, Ibanez F, Banerjee R, El-Mohtar C, Stelinski LL, Bonning BC. 2023. Cry1Ba1-mediated toxicity of transgenic Bergera koenigii and Citrus sinensis to the Asian citrus psyllid Diaphorina citri. Front Insect Sci 3:1125987. doi:10.3389/finsc.2023.1125987. PMC10926525 38469526

[12] Jerga A, Chen D, Zhang C, Fu J, Kouadio JL, Wang Y, Duff SM, Howard JE, Rydel TJ, Evdokimov AG, Ramaseshadri P, Evans A, Bolognesi R, Park Y, Haas JA. 2016. Mechanistic insights into the first Lygus-active beta-pore forming protein. Arch Biochem Biophys 600:1–11. doi:10.1016/j.abb.2016.03.016. 27001423

[13] Ravanfar SA, Achor DS, Killiny N, Shilts T, Chen Y, El-Mohtar C, Stelinski LL, Bonning BC, Orbovic V. 2022. Genetic modification of Bergera koenigii for expression of the bacterial pesticidal protein Cry1Ba1. Front Plant Sci 13:899624. doi:10.3389/fpls.2022.899624. 35685021 PMC9171844

[14] Lacomel CJ, Dunstone MA, Spicer BA. 2021. Branching out the aerolysin, ETX/MTX-2 and Toxin_10 family of pore forming proteins. J Invertebr Pathol 186:107570. doi:10.1016/j.jip.2021.107570. 33775676

[15] Xu C, Chinte U, Chen L, Yao Q, Meng Y, Zhou D, Bi LJ, Rose J, Adang MJ, Wang BC, Yu Z, Sun M. 2015. Crystal structure of Cry51Aa1: a potential novel insecticidal aerolysin-type beta-pore-forming toxin from Bacillus thuringiensis. Biochem Biophys Res Commun 462:184–189. doi:10.1016/j.bbrc.2015.04.068. 25957471

[16] Wenninger EJ, Stelinski LL, Hall DG. 2009. Roles of olfactory cues, visual cues, and mating status in orientation of Diaphorina citri Kuwayama (Hemiptera: Psyllidae) to four different host plants. Environ Entomol 38:225–234. doi:10.1603/022.038.0128. 19791618

[17] Sjolund RD. 1997. The phloem sieve element: a river runs through it. Plant Cell 9:1137–1146. doi:10.1105/tpc.9.7.1137. 12237379 PMC156986

[18] Fukuda A, Fujimaki S, Mori T, Suzui N, Ishiyama K, Hayakawa T, Yamaya T, Fujiwara T, Yoneyama T, Hayashi H. 2005. Differential distribution of proteins expressed in companion cells in the sieve element-companion cell complex of rice plants. Plant Cell Physiol 46:1779–1786. doi:10.1093/pcp/pci190. 16120685

[19] Oparka KJ, Turgeon R. 1999. Sieve elements and companion cells: traffic control centers of the phloem. Plant Cell 11:739–750. doi:10.2307/3870896. 10213790 PMC144213

[20] Ishiwatari Y, Fujiwara T, McFarland KC, Nemoto K, Hayashi H, Chino M, Lucas WJ. 1998. Rice phloem thioredoxin h has the capacity to mediate its own cell-to-cell transport through plasmodesmata. Planta 205:12–22. doi:10.1007/s004250050291. 9599802

[21] Fouquaert E, Hanton SL, Brandizzi F, Peumans WJ, Van Damme EJ. 2007. Localization and topogenesis studies of cytoplasmic and vacuolar homologs of the Galanthus nivalis agglutinin. Plant Cell Physiol 48:1010–1021. doi:10.1093/pcp/pcm071. 17567639

[22] Panneerselvam S, Mishra R, Berry C, Crickmore N, Bonning BC. 2022. BPPRC database: a web-based tool to access and analyse bacterial pesticidal proteins. Database (Oxford) 2022:baac022. doi:10.1093/database/baac022. 35396594 PMC9216523

[23] de Oliveira Dorta S, Borges Attilio L, Zanuzo Zanardi O, Spotti Lopes JR, Machado MA, Freitas-Astua J. 2023. Genetic transformation of 'Hamlin' and 'Valencia' sweet orange plants expressing the cry11A gene of Bacillus thuringiensis as an additional tool for the management of Diaphorina citri (Hemiptera: Liviidae). J Biotechnol 368:60–70. doi:10.1016/j.jbiotec.2023.04.007. 37088156

[24] Carriere Y, Fabrick JA, Tabashnik BE. 2016. Can pyramids and seed mixtures delay resistance to Bt crops? Trends Biotechnol 34:291–302. doi:10.1016/j.tibtech.2015.12.011. 26774592

[25] de Bortoli CP, Jurat-Fuentes JL. 2019. Mechanisms of resistance to commercially relevant entomopathogenic bacteria. Curr Opin Insect Sci 33:56–62. doi:10.1016/j.cois.2019.03.007. 31358196

[26] Gassmann AJ, Reisig DD. 2023. Management of insect pests with Bt crops in the United States. Annu Rev Entomol 68:31–49. doi:10.1146/annurev-ento-120220-105502. 36170641

[27] Hall DG, Hentz MG. 2011. Seasonal flight activity by the Asian citrus psyllid in east central Florida. Entomol Exp Appl 139:75–85. doi:10.1111/j.1570-7458.2011.01108.x.

[28] Lewis-Rosenblum H, Martini X, Tiwari S, Stelinski LL. 2015. Seasonal movement patterns and long-range dispersal of Asian citrus psyllid in Florida citrus. J Econ Entomol 108:3–10. doi:10.1093/jee/tou008. 26470097

[29] Kobori Y, Nakata T, Ohto Y, Takasu F. 2011. Dispersal of adult Asian citrus psyllid, Diaphorina citri Kuwayama (Homoptera: Psyllidae), the vector of citrus greening disease, in artificial release experiments. Appl Entomol Zool 46:27–30. doi:10.1007/s13355-010-0004-z.

[30] Behlau F, Belasque J, Filho AB, Graham JH, Leite RP, Gottwald TR. 2008. Copper sprays and windbreaks for control of citrus canker on young orange trees in southern Brazil. Crop Protection 27:807–813. doi:10.1016/j.cropro.2007.11.008.

[31] Gottwald TR, Timmer LW. 1995. Efficacy of windbreaks in reducing the spread of citrus canker caused by Xanthomonas campestris pv. citri. Trop Agric 72:194–201.

[32] Beloti VH, Santos F, Alves GR, Bento JMS, Yamamoto PT. 2017. Curry leaf smells better than citrus to females of Diaphorina citri (Hemiptera: Liviidae). Arthropod-Plant Interact 11:709–716. doi:10.1007/s11829-017-9524-6.

[33] Tomaseto AF, Krugner R, Lopes JRS. 2016. Effect of plant barriers and citrus leaf age on dispersal of Diaphorina citri (Hemiptera: Liviidae). J Appl Entomol 140:91–102. doi:10.1111/jen.12249.

[34] Eduardo WI, Silva AC, Volpe HXL, Alquezar B, Pena L, Miranda MP. 2023. Push-pull and kill strategy for Diaphorina citri control in citrus orchards. Entomologia Exp Applicata 171:287–299. doi:10.1111/eea.13273.

[35] Beloti VH, Alves GR, Coletta-Filho HD, Yamamoto PT. 2018. The Asian citrus psyllid host Murraya koenigii is immune to citrus huanglongbing pathogen 'Candidatus Liberibacter asiaticus.' Phytopathol 108:1089–1094. doi:10.1094/PHYTO-01-18-0012-R. 29648945

[36] Yan H, Zeng J, Zhong G. 2015. The push–pull strategy for citrus psyllid control. Pest Manag Sci 71:893–896. doi:10.1002/ps.3915. 25256398

[37] Pierre MO, Salvatierra-Miranda J, Rivera MJ, Etxeberria E, Gonzalez P, Vincent CI. 2021. White and red-dyed kaolin particle films reduce Asian citrus psyllid populations, delay huanglongbing infection, and increase citrus growth. Crop Protection 150:105792. doi:10.1016/j.cropro.2021.105792.

[38] Hall DG, Lapointe SL, Wenninger EJ. 2007. Effects of a particle film on biology and behavior of Diaphorina citri (Hemiptera: Psyllidae) and its infestations in citrus. J Econ Entomol 100:847–854. doi:10.1603/0022-0493(2007)100[847:eoapfo]2.0.co;2. 17598547

[39] Bachman PM, Ahmad A, Ahrens JE, Akbar W, Baum JA, Brown S, Clark TL, Fridley JM, Gowda A, Greenplate JT, Jensen PD, Mueller GM, Odegaard ML, Tan J, Uffman JP, Levine SL. 2017. Characterization of the activity spectrum of MON 88702 and the plant-incorporated protectant Cry51Aa2.834_16. PLoS One 12:e0169409. doi:10.1371/journal.pone.0169409. 28072875 PMC5224830

[40] Farmer DR, Edrington TC, Kessenich CR, Wang C, Petrick JS. 2017. Improving insect control protein activity for GM crops: a case study demonstrating that increased target insect potency can be achieved without impacting mammalian safety. Regul Toxicol Pharmacol 89:155–164. doi:10.1016/j.yrtph.2017.07.020. 28751263

[41] Boss A, Romeis J, Meissle M. 2022. Prey-mediated effects of mCry51Aa2-producing cotton on the predatory nontarget bug Orius majusculus (Reuter). Insect Sci doi:10.1111/1744-7917.13143. 36385458

[42] Dutt M, Ananthakrishnan G, Jaromin MK, Brlansky RH, Grosser JW. 2012. Evaluation of four phloem-specific promoters in vegetative tissues of transgenic citrus plants. Tree Physiol 32:83–93. doi:10.1093/treephys/tpr130. 22228816

[43] Miyata LY, Harakava R, Stipp LC, Mendes BM, Appezzato-da-Gloria B, de Assis Alves Mourao Filho F. 2012. GUS expression in sweet oranges (Citrus sinensis L. Osbeck) driven by three different phloem-specific promoters. Plant Cell Rep 31:2005–2013. doi:10.1007/s00299-012-1312-2. 22801867

[44] Orbović V, Grosser JW. 2015. Citrus transformation using juvenile tissue explants. Methods Mol Biol 1224:245–257. doi:10.1007/978-1-4939-1658-0_20. 25416263

[45] Orbović V, Grosser JW. 2006. Citrus: sweet orange (*Citrus sinensis* L. Osbeck ‘Valencia’) and Carrizo citrange [*Citrus sinensis* (L.) Osbeck x *Poncirus trifoliata* (L.) Raf.], p 177–189. *In* Wang K (ed), Agrobacterium protocols: methods in molecular biology. Humana Press Inc., New York, NY.

[46] Dutt M, Li ZT, Dhekney SA, Gray DJ. 2012. Co-transformation of grapevine somatic embryos to produce transgenic plants free of marker genes. Methods Mol Biol 847:201–213. doi:10.1007/978-1-61779-558-9_17. 22351010

[47] Livak KJ, Schmittgen TD. 2001. Analysis of relative gene expression data using real-time quantitative PCR and the 2(-delta delta C(T)) method. Methods 25:402–408. doi:10.1006/meth.2001.1262. 11846609

[48] Bradford MM. 1976. A rapid and sensitive method for the quantitation of microgram quantities of protein utilizing the principle of protein-dye binding. Anal Biochem 72:248–254. doi:10.1016/0003-2697(76)90527-3. 942051

[49] Chen XD, Neupane S, Gossett H, Pelz-Stelinski KS, Stelinski LL. 2021. Insecticide rotation scheme restores insecticide susceptibility in thiamethoxam-resistant field populations of Asian citrus psyllid, Diaphorina citri Kuwayama (Hemiptera: Liviidae), in Florida. Pest Manag Sci 77:464–473. doi:10.1002/ps.6039. 32770656

[50] Johnston N, Stansly P, Stelinski LL. 2019. Secondary hosts of Asian citrus psyllid, Diaphorina citri Kuwayama: survivorship and preference. J Appl Entomol 143:921–928. doi:10.1111/jen.12673.

[51] Spurr AR. 1969. A low-viscosity epoxy resin embedding medium for electron microscopy. J Ultrastruct Res 26:31–43. doi:10.1016/s0022-5320(69)90033-1. 4887011

[52] Reynolds ES. 1963. The use of lead citrate at high pH as an electron-opaque stain in electron microscopy. J Cell Biol 17:208–212. doi:10.1083/jcb.17.1.208. 13986422 PMC2106263

[53] Kaplan EL, Meier P. 1958. Nonparametric estimation from incomplete observations. J Am Stat Assoc 53:457–481. doi:10.1080/01621459.1958.10501452.

[54] R Core Team. 2020. RStudio: integrated development for R. RStudio, Boston, MA.

