# Supplementary material for: Bacterial Pesticidal Protein Mpp51Aa1 Delivered via Transgenic Citrus Severely Impacts the Fecundity of Asian Citrus Psyllid, Diaphorina citri
Source: Appl Environ Microbiol. 2023 Jul 17;89(8):e00723-23. doi: 10.1128/aem.00723-23 (PMC10467345; doi:10.1128/aem.00723-23)

Supplementary Information

Figure S1. Mpp51Aa1 sequences used for vector construction. Coding sequences were modified according to citrus codon preference (grapefruit). **A)** Coding sequence for Mpp51Aa1 with GNA secretory signal peptide (in bold) inserted into psM3. **B)** Mpp51Aa1 coding sequence inserted into pM4. Primers used for confirmation of presence in the T-DNA are highlighted: primer set 1 in bold italics, 2 underlined, 3 in bold underlined. See also Tables S1 and S2).

A. psM3

ATGGCTAAGGCTTCTCTTTTGATTTTGGCTGCTATTTTTCTTGGAGTTATTACTCCTTCATGTTTG
TCTGCTATTCTGGATCTCAAGTCACTGGTTCTCAACGCTATTAACTATTGGGGACCTAAGAACAAC
AACGGCATTCAGGGTGGAGATTTCGGATATCCTATTTCTGAAAAGCAAATTGATACTTCTATTATTACATCAACT
CATCCTAGACTTATTCCACACGATTTGACTATTCCACAAAATCTTGAGACAATTTTTACTACAACTCAGGTTCTT
ACTAATAACACAGATTTGCAACAGTCTCAGACTGTTTCATTTGCTAAGAAAACAACTACAACTACATCAACTTCT
ACTACAAATGGTTGGACAGAAGGTGGAAAGATTTCTGATACATTGGAAGAGAAAGTTTCAGTTTCTATTCCTTTT
ATTGGTGAAGGTGGTGGAAAAAATTCAACTACAATTGAGGCTAACTTCGCTCATAATTCATCTACTACAACTTTC
CAACAGGCTTCTACTGATATTGAGTGGAATATTTCACAACCAGTTCTTGTTCCTCCAAGAAAACAGGTTGTTGCT
ACTTTGGTTATTATGGGAGGTAATTTTACAATTCCTATGGATCTTATGACAACTATTGATTCTACTGAGCACTAT
TCAGGATATCCAATTTTGACATGGATTTCATCTCCTGATAACTCTTACAATGGTCCTTTTATGTCATGGTACTTT
GCTAATTGGCCTAATCTTCCATCTGGATTTGGTCCTCTTAATTCAGATAACACAGTTACTTACACAGGATCAGTT
GTTTCTCAAGTTTCAGCTGGTGTTTATGCTACTGTTAGGTTTGATCAGTATGATATTCATAATCTTAGAACTATT
GAGAAGACATGGTATGCTAGGCATGCTACATTGCACAATGGTAAAAAGATTTCTATTAACAATGTG
ACAGAGATGGCACCTACAAGCCCTATTAAAACAAAC**TAATAG**

B. pM4

ATGGCTATTCTGGATCTCAAGTCACTGGTTCTCAACGCTATTAACTATTGGGGACCTAAGAACAAC
AAC***GGCATTCAGGGTGGAGATTT***CGGATATCCTATTTCTGAAAAGCAAATTGATACTTCTATTATTACATCAA***CT***
CATCCTAGACTTATTCCACACGATTTGACTATTCCACAAAATCTTGAGACAATTTTTACTACAACTCAGGTTCTT
ACTAATAACACAGATTTGCAACAGTCTCAGACTGTTTCATTTGCTAAGAAAACAACTACAACTACATCAACTTCT
ACTACAAATGGTTGGACAGAAGGTGGAAAGATTTCTGATACATTGGAAGAGAAAGTTTCAGTTTCTATTCCTTTT
<u>ATTGGTGAAGGTGGTGGAAA</u>AAATTCAACTACAATTGAGGCTAACTTCGCTCATAATTCATCTACTACAA<u>CTTTC</u>
<u>CAACAGGCTTCTACTGA</u>TATTGAGTGGAATATTTCACAACCAGTTCTTGTTCCTCCAAGAAAACAGGTTGTTGCT
ACTTTGGTTATTATGGGAGGTAATTTTACAATTCCTATGGATCTTATGACAACTATTGATTCTACTGAGCACTAT
TCAGGATATCCAATTTTGACATGGATTTCATCTCCTGATAACTCTTACAATGGTCCTTTTATGTCATGGTACTTT
GCTAATTGGCCTAATCTTCCATCTGGATTTGGTCCTCTTAATTCAGATAACACAGTTACTTACACAGGATCAGTT
GTTT<u>**CTCAAGTTTCAGCTGGTGTTTATG**</u>CTACTGTTAGGTTTGATCAGTATGATATTCATAATCTTAGAACTATT
<u>**GAGAAGACATGGTATGCTAGGC**</u>ATGCTACATTGCACAATGGTAAAAAGATTTCTATTAACAATGTGACAGAGATG
GCACCTACAAGCCCTATTAAAACAAAC**TAATAG**

Table S1. Primers used for PCR in this study. The sequences and positions of the three sets of primers (sets 1-3) used to confirm the presence of the Mpp51Aa1 sequence in the T-DNA are provided. See also Figure S1 for locations within the Mpp51Aa1 sequence, and Table S2 for results of the analysis.

Description	Name	Sequence	Position in *mpp51aa1*
1F	M-1768	GGCATTCAGGGTGGAGATTT	70-89
1R	M-1769	CGTGTGGAATAAGTCTAGGATGAG	163-140
2F	M1770	CTCAAGTTTCAGCTGGTGTTTATG	746-769
2R	M1771	GCCTAGCATACCATGTCTTCTC	838-817
3F	M1772	ATTGGTGAAGGTGGTGGAAA	367-386
3R	M1773	TCAGTAGAAGCCTGTTGGAAAG	458-437

Table S2. Confirmation of *mpp51aa1* presence in representative transgenic plants. Quantitative PCR analysis with three sets of primers confirming the presence of *mpp51aa1* in transgenic plants. Data for control plants (WT) and the qPCR negative control (H_2O) are also shown.

	**Primer set-1**		**Primer set-2**		**Primer set-3**		**ACT**	
**Plant**	**Ct**	**Tm**	**Ct**	**Tm**	**Ct**	**Tm**	**Ct**	**Tm**
H_2O	U	NA	U	NA	U	NA	U	N/A
VAL WT	U	NA	U	NA	U	NA	22.64±0.27	77.14±0.19
**VAL368-011**	22.38±0.12	72.52±0.19	22.79±0.18	73.22±0.11	22.60±0.35	74.40±0.00	22.15±0.22	77.72±0.19
**VAL368-012**	22.89±0.04	72.13±0.19	23.32±0.34	72.81±0.14	22.91±0.08	74.06±0.27	22.53±0.11	77.33±0.19
**VAL369-02**	22.31±0.07	72.71±0.00	22.56±0.13	73.29±0.19	23.55±0.23	74.38±0.11	23.32±0.03	77.65±0.11
**VAL400-01**	23.28±0.10	72.77±0.11	23.50±0.09	73.48±0.00	22.67±0.24	74.57±0.11	22.67±0.03	77.59±0.22
**VAL400-02**	22.39±0.05	72.90±0.00	22.61±0.12	73.48±0.00	22.16±0.24	74.64±0.00	22.73±0.16	77.72±0.00
**VAL409-01**	23.01±0.06	72.84±0.11	23.28±0.04	72.42±0.11	23.53±0.25	74.44±0.00	22.72±0.03	77.59±0.11

VAL, Valencia sweet orange; ACT, actin; Ct, cycle threshold; Tm, primer melting temperature; U, undetermined; NA, not applicable.

Figure S2. Western blot detection of Mpp51Aa1 in transgenic Duncan and Valencia plants. Western blot analyses for detection of Mpp51Aa1 expressed by transgenic Duncan plants, and Valencia where indicated. Bands of the expected size for Mpp51Aa1 are indicated by arrows within the green box. Degradation products of ~16-17 kDa are boxed within red dashed lines. Mpp51Aa1 of the expected size was detected in 15 of the Duncan lines when expressed without the signal peptide (pM4), while no full length Mpp51Aa1 was detected on expression with the signal peptide (psM3). A summary of results is provided in Tables 2 and S3. M, molecular mass markers shown at right (kD); Control, wild type Duncan. Proteins were detected using a 1:2,500 dilution of anti-Mpp51Aa1 and 1:2,500 dilution of anti-rabbit IgG with 20 µg protein loaded per lane (apart from the positive control Mpp51Aa1) as described in Methods.

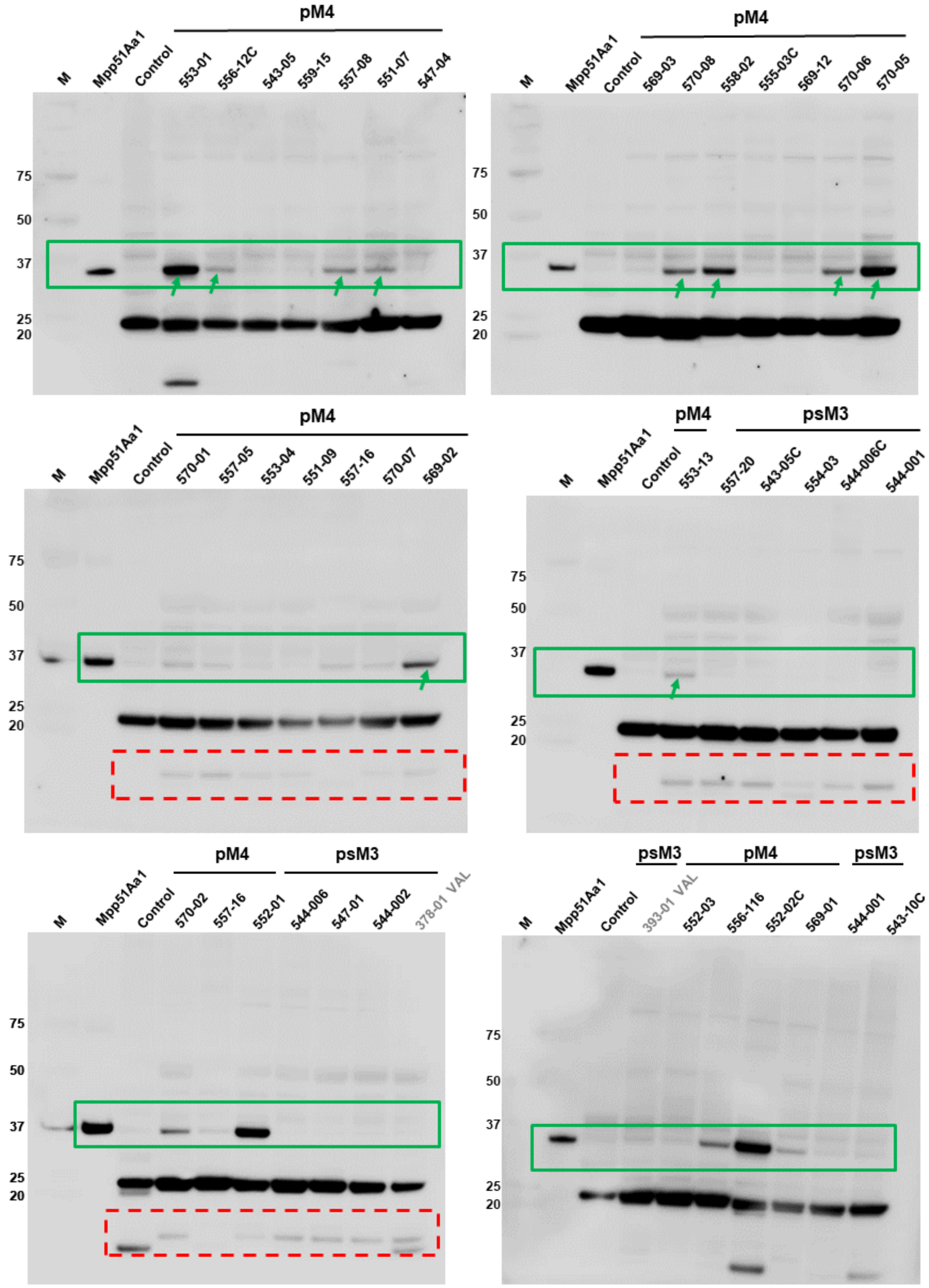

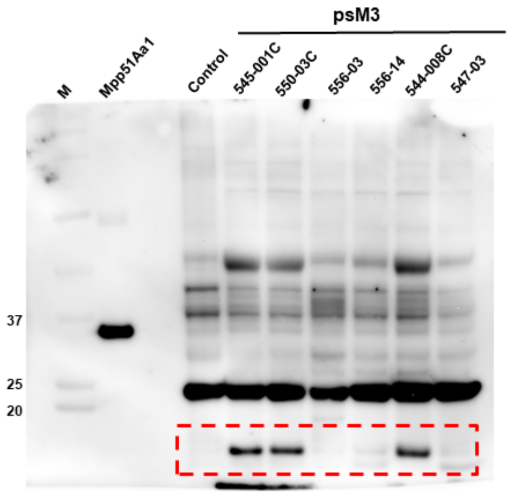
psM3
M
Mpp51Aa1
Control
545-001C
550-03C
556-03
556-14
544-008C
547-03
37
25
20

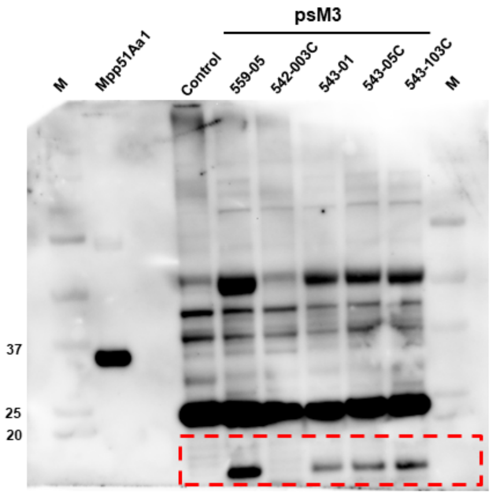
psM3
M
Mpp51Aa1
Control
559-05
542-003C
543-01
543-05C
543-103C
M
37
25
20

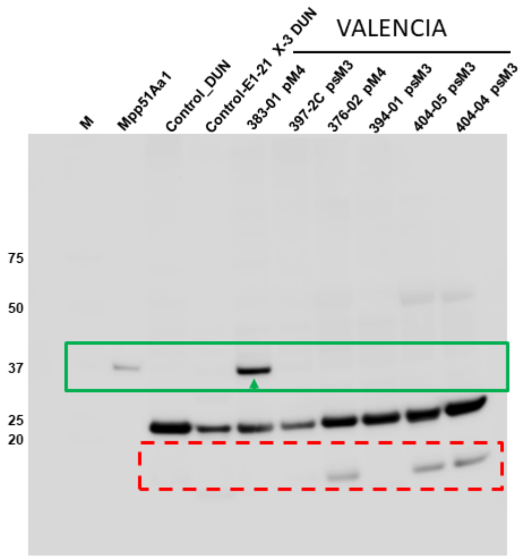
VALENCIA
M
Mpp51Aa1
Control_DUN
Control-E1-21 X-3 DUN
383-01 pM4
397-2C psM3
376-02 pM4
394-01 psM3
404-05 psM3
404-04 psM3
75
50
37
25
20

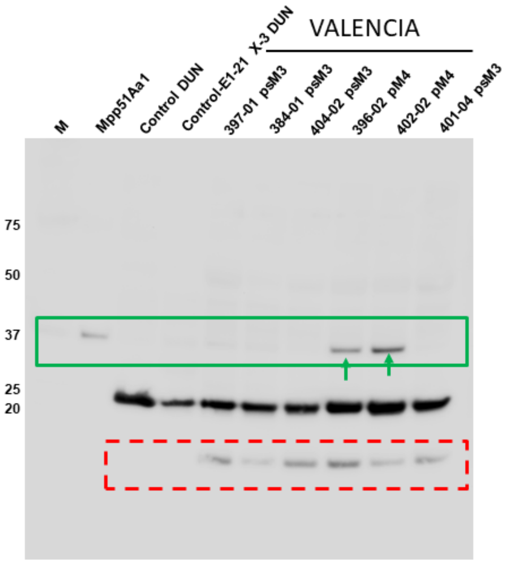
VALENCIA
M
Mpp51Aa1
Control DUN
Control-E1-21 X-3 DUN
397-01 psM3
384-01 psM3
404-02 psM3
396-02 pM4
402-02 pM4
401-04 psM3
75
50
37
25
20

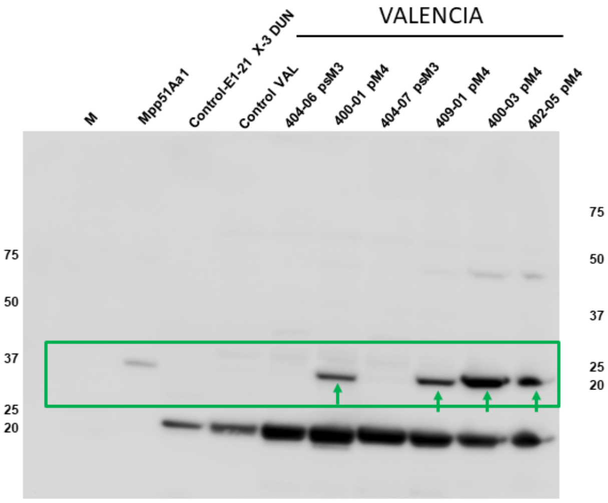
VALENCIA
M
Mpp51Aa1
Control-E1-21 X-3 DUN
Control VAL
404-06 psM3
400-01 pM4
404-07 psM3
409-01 pM4
400-03 pM4
402-05 pM4
75
50
37
25
20

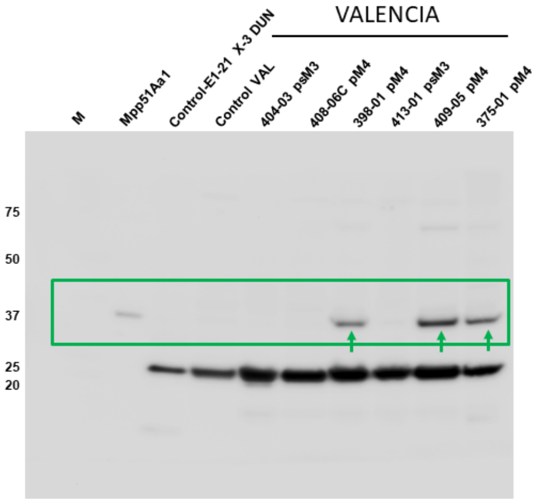
VALENCIA
M
Mpp51Aa1
Control-E1-21 X-3 DUN
Control VAL
404-03 psM3
408-06C pM4
398-01 pM4
413-01 psM3
409-05 pM4
375-01 pM4
75
50
37
25
20

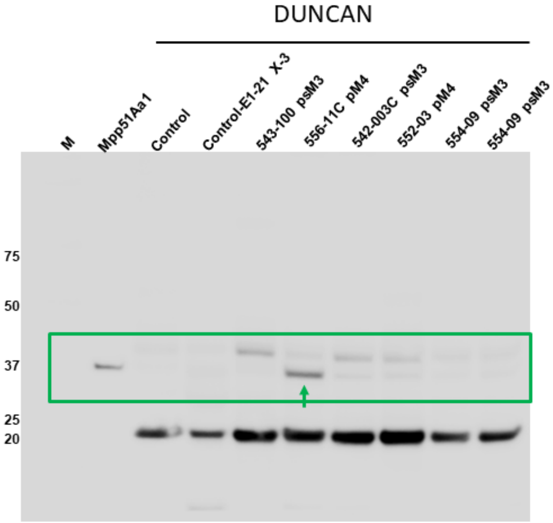
DUNCAN
M
Mpp51Aa1
Control
Control-E1-21 X-3
543-100 psM3
556-11C pM4
542-003C psM3
552-03 pM4
554-09 psM3
554-09 psM3
75
50
37
25
20

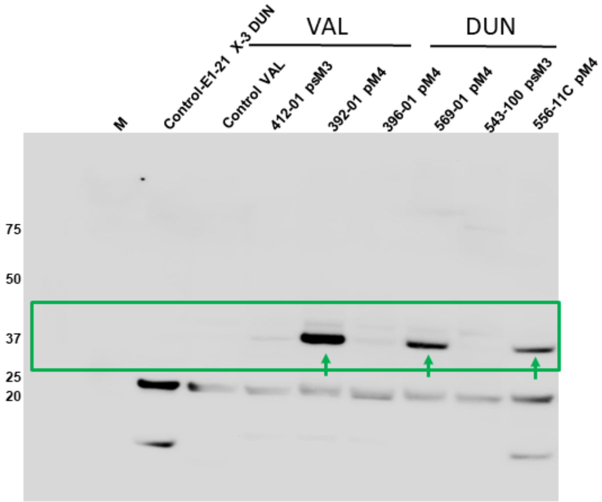
VAL
DUN
M
Control-E1-21 X-3 DUN
Control VAL
412-01 psM3
392-01 pM4
396-01 pM4
569-01 pM4
543-100 psM3
556-11C pM4
75
50
37
25
20

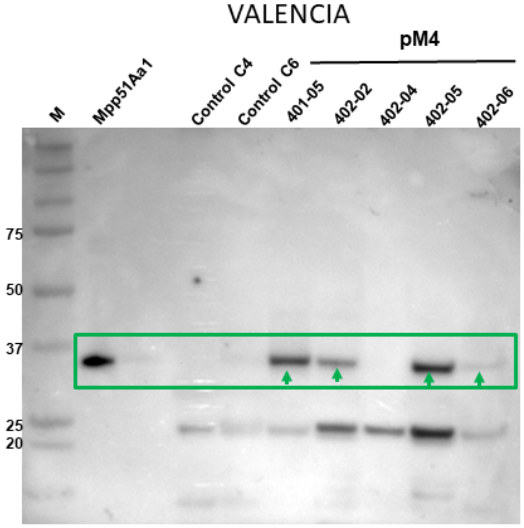
VALENCIA
pM4
M
Mpp51Aa1
Control C4
Control C6
401-05
402-02
402-04
402-05
402-06
75
50
37
25
20

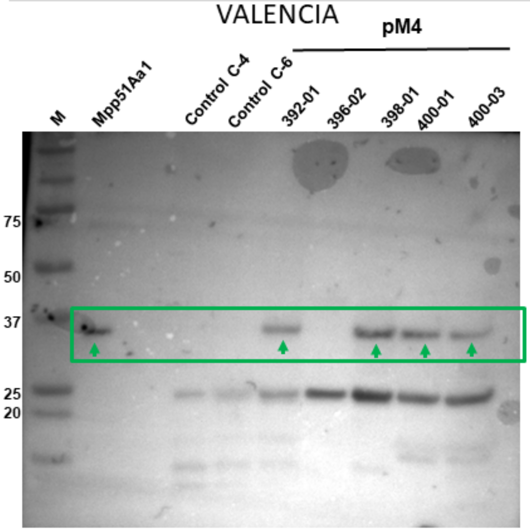
VALENCIA
pM4
M
Mpp51Aa1
Control C-4
Control C-6
392-01
396-02
398-01
400-01
400-03
75
50
37
25
20

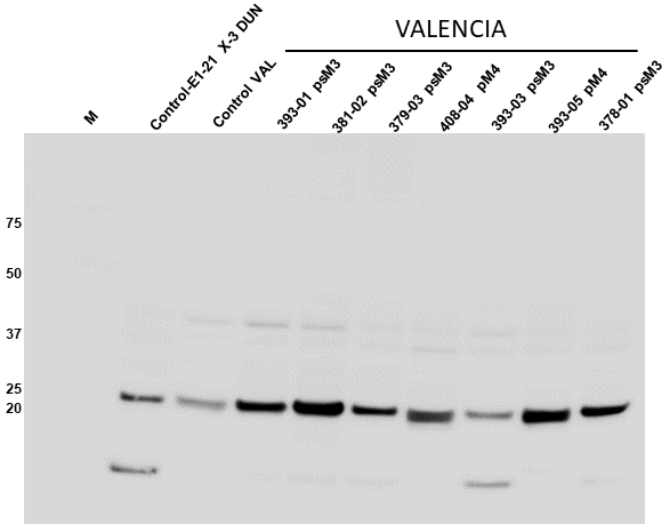
VALENCIA
M
Control-E1-21 X-3 DUN
Control VAL
393-01 psM3
381-02 psM3
379-03 psM3
408-04 pM4
393-03 psM3
393-05 pM4
378-01 psM3
75
50
37
25
20

Table S3. Transcript levels of *mpp51aa1* and the absence of Mpp51Aa1 protein of the expected size as determined by western blot analysis in psM3 transgenic Duncan and Valencia plants.

	Duncan	Relative transcript level	Protein expression	Valencia	Relative transcript level	Protein expression
1	543-01	630.06	-d	378-01	12239.09	-d
2	543-05c	156.9	-d	379-03	4374.52	-
3	544-008c	2023.94	-d	381-02	1951.18	-
4	545-10	136.6	-d	384-01	2698.97	-d
5	547-01	0.4	-	393-01	1314.17	-
6	547-03	292.5	-d	393-03	206.05	-
7	547-04	813.23	-d	394-01	796.76	-
8	554-03	104.4	-d	397-01	1470.26	-d
9	554-09	738.65	-	397-2c	2079.74	-
10	556-03	1053.34	-	412-01	2014.19	-
11	556-04	966.55	-d	404-07	652.46	-
12	556-14	3742.89	-d	413-01	171.33	-
13	557-10	5374.4	-d	404-04	16.2	-d
14	557-20	359.16	-d	404-02	2421.17	-d
15	559-05	2925.21	-d	404-05	2094.21	-d
16	542-001	57.5	-	401-04	6131.73	-d
17	542-002c	307.1	-d	404-03	80.5	-
18	542-003c	0	-	404-06	1604.23	-
19	543-100	58.26	-	C4	0	-
20	543-102c	416.2	-d	C6	0	-
21	544-001	0	-d			
22	544-002	164.9	-d			
23	544-006c	151.43	-d			
24	545-001c	30.86	-d			
25	550-03c	9.4	-d			
26	554-01	5826.97	-d			

d, Mpp51Aa1-immunoreactive bands at a smaller size than expected present, indicative of degradation. Western blots are provided in Figure S2.

Figure S3. Expression of Mpp51Aa1 did not impact adult *D. citri* host selection in choice tests between transgenic and control Duncan plants. All Duncan plants were of similar age and height. The data represent three independent replicate experiments with no significant difference detected (chi-square = 0.8554; *df* is 1; *p* = 0.357862).

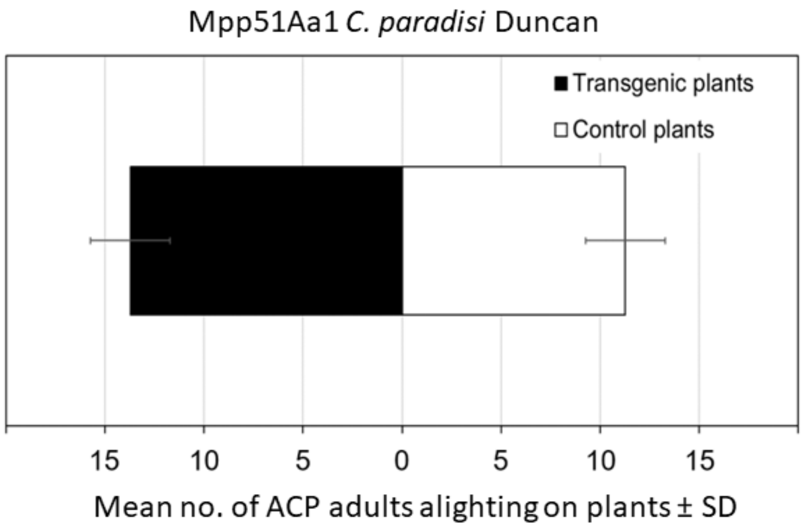

Figure S4. The flush status of Mpp51Aa1-expressing transgenic Duncan does not impact survival of adult *D. citri*. To assess with low adult psyllid mortality on transgenic Duncan plants was associated with lack of new leaf growth (flush), bioassays were conducted on plants, with and without flush. No statistical differences were detected in adult survival over the course of the assay, with three independent replicates (Log-Rank test for pairwise comparisons).

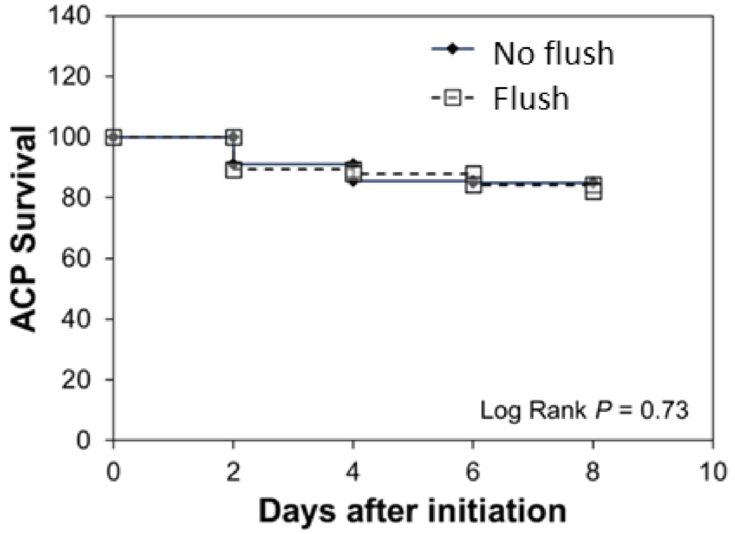

Figure S5. Damage to the gut epithelium of adult *Diaphorina citri* was consistent with mortality observed. Transmission electron micrographs showing *D. citri* gut epithelia following feeding on control or transgenic Mpp51Aa1-expressing *C. paradisi* cv. Duncan. The microvilli for a subset of psyllids fed on transgenic plants were thin, short and / or disrupted relative to the intact microvilli seen in psyllids fed on control plants. L, gut lumen; BM, basement membrane; MV, microvilli, sMV short microvilli, **area of thin microvilli. Scale bars, 1µm.

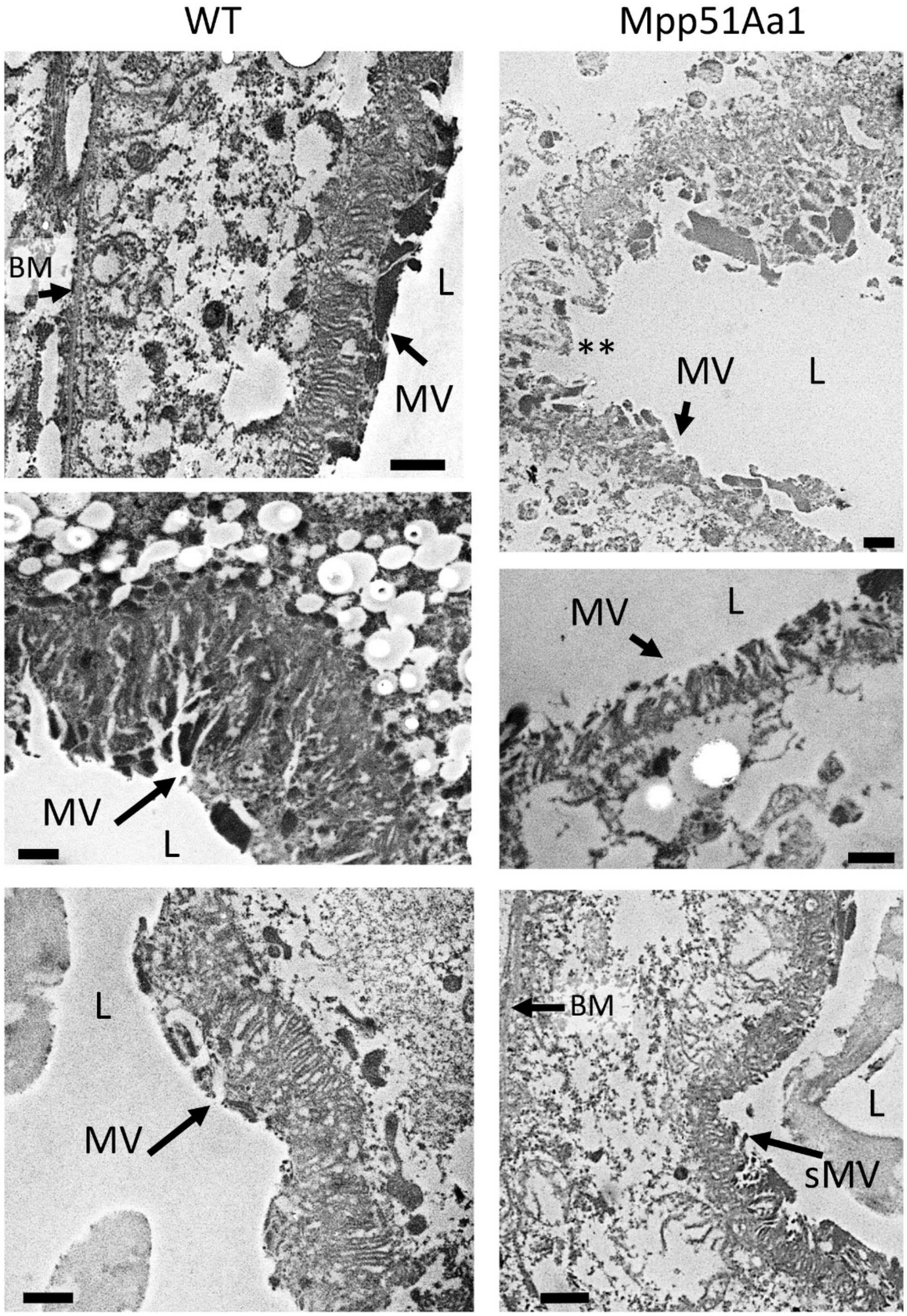

Supplement: Supplemental file 1 — Fig. S1 to S5 and Tables S1 to S3. Download aem.00723-23-s0001.pdf, PDF file, 2.9 MB [file aem.00723-23-s0001.pdf]
